# Selective Enrichment of Clenbuterol onto Molecularly Imprinted Polymer Microspheres with Tailor-made Structure and Oxygen Functionalities

**DOI:** 10.3390/polym11101635

**Published:** 2019-10-10

**Authors:** Xiangyun Zhao, Yuliang Mai, Dongchu Chen, Min Zhang, Huawen Hu

**Affiliations:** 1 Guangdong Provincial Key Laboratory of Industrial Surfactant, Guangdong Research Institute of Petrochemical and Fine Chemical Engineering, Guangzhou 510006, China; xiangyunzhao2009@sina.com; 2 School of Materials Science and Energy Engineering, Foshan University, Foshan, Guangdong 528000, China; cdcever@163.com (D.C.); zhangmin@gic.ac.cn (M.Z.)

**Keywords:** molecularly imprinted polymers, polymer microspheres, Pickering emulsion, oxygen functionalities, adsorption, selectivity

## Abstract

The noxious clenbuterol misapplied as the feed additive has posed an enormous threat to humans who actively rely on the food chains with high potential of contamination by clenbuterol, such as pork and beef. It is, therefore, highly desirable to develop novel materials and strategies for dealing with the clenbuterol. Herein, functional polymer microspheres prepared by Pickering emulsion polymerization were explored for the selective enrichment of the clenbuterol, and their structure and oxygen functionalities could be tailor-made by a molecular imprinting process. The clenbuterol imprinting was adequately demonstrated to not only increase the particle size (~52 nm vs. ~42 nm) and create cavities for the accommodation of the clenbuterol molecules, but also reduce the oxygen functionalities of the resulting molecularly imprinted polymer microspheres (MIPMs) by approximately 4 at.%, which is believed to correlate with the high specificity of the MIPMs. Various characterization methods were employed to evidence these findings, including scanning electron microscopy, BET measurements, Fourier transform infrared spectroscopy, X-ray photoelectron spectroscopy, and elemental mapping examination. More importantly, the MIPMs showed a markedly superior enrichment capability towards clenbuterol to the counterpart, that is, non-molecularly imprinted polymer microspheres (NIPMs). Compared to the NIPMs without specificity for clenbuterol, the MIPMs exhibited an impressive selectivity to clenbuterol, with the relative selectivity coefficient (*k′*) values largely exceeding 1, thus corroborating that the useful molecular imprinting led to the generation of the binding sites complementary to the clenbuterol molecule in the size and functionalities. The MIPMs were also employed as the stationary phase to fabricate molecularly imprinting solid-phase extraction column, and the spike recovery was demonstrated to be not significantly decreased even after nine cycles. Furthermore, the reliability of the method was also evidenced through the comparison of the MIPMs prepared from different batches.

## 1. Introduction

Clenbuterol, a β_2_-adrenoceptor agonist, has raised worldwide concern due to its widespread applications as remedial drugs for the treatment of bronchial diseases (e.g., asthma, pulmonary emphysema, and chronic bronchitis) [1], and even more extensive attention is paid to its function as “lean meat powder”, which can stimulate the transformation of fat tissue to muscular tissue in livestock [2]. Since lean meat is the more favorable food relative to the fat, many illegal producers are motivated to misapply clenbuterol for the stimulation of lean meat growth. However, clenbuterol is highly toxic to humans, with a long half-life period, which may lead to acute arrhythmic events [3,4]. Even worse, the threat of the clenbuterol to human life safety can be generated through the favorite food chain such as the pork and beef [4] although the application of clenbuterol is strictly prohibited in many countries, including the United States, China, most European countries, etc. [4], and food poisoning due to clenbuterol are frequently reported [5]. Therefore, there is an urgent need for the development of new materials and techniques for dealing with noxious clenbuterol.

Before the monitoring of clenbuterol, enrichment processing is usually needed to obtain more accurate results and lower limits of detection by the subsequent instrumental investigation of the pre-concentrated clenbuterol (e.g., high-performance liquid chromatography (HPLC), liquid chromatography-mass spectrometry (LC-MS), and gas chromatography-mass spectrometry (GC-MS) [4,5,6]). Therefore, enrichment processing plays a vital role in the handling of organic wastes in the analytical fields [7,8]. Adsorption is one of the most commonly used approaches for the enrichment of various contaminants including clenbuterol [9], organic dyes [10], inorganic metal ions [11], etc. [7,12], and many kinds of adsorbents have been explored, such as polymer microspheres [13,14], polymer fibers [15], polymer hydrogels [16], waste biomasses [17], activated carbon [18], carbon nanotubes [19], graphene [20], and silica nanoparticles [21]. However, these materials can bind a range of contaminants in a nonspecific manner [22], e.g., polymer microspheres exhibit nonspecific adsorption performance towards a spectrum of dyes [23]. These adsorbents are thus unsuitable for the enrichment of clenbuterol due to their lack of specificity.

Molecular imprinting technologies are widely employed to render diverse materials selective to a target species [2,24]. Molecularly imprinted polymers (MIPs) are examples of considerable concern for various applications including clinical diagnostics [25], cell and tissue imaging [26], solid-phase extraction [27], catalysis [28], sensing [29], drug loading and delivery [30], etc. [31], and a broad range of analytes has been tackled with MIPs, such as enrofloxacin [29], malachite green [32], ibuprofen [33], dopamine [34] and clenbuterol [24,35,36,37]. The useful molecular imprinting treatment lies in its capability of creating binding sites at the surface or within the matrix of polymeric materials complementary to the imprint species in the size, shape, functionalities, and coordination geometries [38,39]. For more information on the MIPs, one can refer to recently published review papers [37,40,41,42].

Although clenbuterol-imprinted polymers have been researched over these years, there still lacks an in-depth comparison between MIPs and non-molecularly imprinted polymers (NIPs) on the specific structural and property variation imparted via the clenbuterol imprinting treatment (e.g., the change in the oxygen functionality concentration endowed by the clenbuterol imprinting). The polymerization in the presence of clenbuterol will alter the solubility of the polymer chains [13], eventually resulting in the size and surface functionalities of MIPs different from NIPs [32]. Besides, the unique structural feature of the clenbuterol template bearing a hydrophobic moiety (–C(CH_3_)_3_) at one end and a hydrophilic moiety (–NH_2_) at the other might also help to improve the stability of the emulsion and consequently the quality of the emulsion polymerization products. The subsequent solvent extraction of clenbuterol leads to the generation of artificial recognition sites being complementary to clenbuterol [43], indicating that the porous structure and the coordination geometries can be tailor-made for the removal of the clenbuterol molecules from the contaminated water.

In this study, we systematically explored the impact of the clenbuterol molecular imprinting on the polymer microspheres with respect to the microstructure, surface functionalities, and adsorption performance towards clenbuterol. Through a comparison between the resulting MIP microspheres (MIPMs) and NIP microspheres (NIPMs), the molecular imprinting effect on the structure and functionalities of MIPMs was clarified. Pickering emulsion polymerization was adopted to synthesize MIPMs rather than the conventional strategies such as bulk polymerization [44], precipitation polymerization [33], and swelling combined with thermal polymerization [45]. Bulk polymerization produces typically monolithic polymer materials that need to be crushed, ground and sieved into powder with the desired range of the particle size. Consequently, this post-treatment procedure is time-consuming and usually leads to irregular particles with a wide distribution of particle size [13]. Precipitation polymerization involves the use of a large amount of the organic solvent, which is undesirable from the environmental viewpoint. Swelling treatment combined with thermal polymerization is multistep, tedious, and time-consuming. By contrast, through the Pickering emulsion polymerization possessing advantages such as emulsion stability and easy operation without further grounding post-treatment, polymer microspheres are readily synthesized with a narrower size distribution. The Pickering emulsion polymerization lies in the utilization of colloidal solid particles with a desired wettability, which results in the formation of oil-in-water or water-in-oil emulsions. They can thus assist in significantly reducing the usage amount of the traditional hazardous organic surfactant for the emulsion preparation, thereby substantially reducing the adverse impact on the environment and humans. Pickering emulsion polymerization has been explored for the preparation of polymer microspheres imprinted with malachite green [32], bisphenols [13], protein [46], λ-cyhalothrin [47] and erythromycin [48]. Nevertheless, no reports can be found on the systematic investigation of the clenbuterol-imprinted polymer microspheres prepared by Pickering emulsion polymerization, e.g., the relationship between their microstructure and surface properties and the adsorption performance toward clenbuterol (including adsorption kinetics, isotherms, thermodynamics, and selectivity, as well as molecularly imprinted solid-phase extraction (MISPE) column applications).

## 2. Experimental

### 2.1. Materials

Fumed silica with a mean diameter of 12 nm (Aerosil 200, 99% purity) was supplied by Evonik Degussa (Evonik Industries AG Inorganic Materials, Hanau, Germany). The initiator, 2,2′-azoisobutyronitrile (AIBN, 99% purity), and the monomer, methacrylic acid (MAA, analytical reagent) were purchased from Tianjin Fucheng Chemical Reagent Factory (Tianjin, China). Clenbuterol, terbutaline, salbutamol, and methyl red were analytical reagents and supplied by Sigma Chemicals Co. Ltd. (Poole, UK). The cross-linking agent, ethylene glycol dimethyl acrylate (EGDMA, 98% purity), was purchased from Aladdin Reagent (Shanghai, China). All of the other chemicals were of the analytical grade and obtained from Tianjin Fucheng Chemical Reagent Factory (Tianjin, China). All of the chemicals were used without further purification unless otherwise stated.

### 2.2. Synthesis of MIPMs

**Figure 1:**
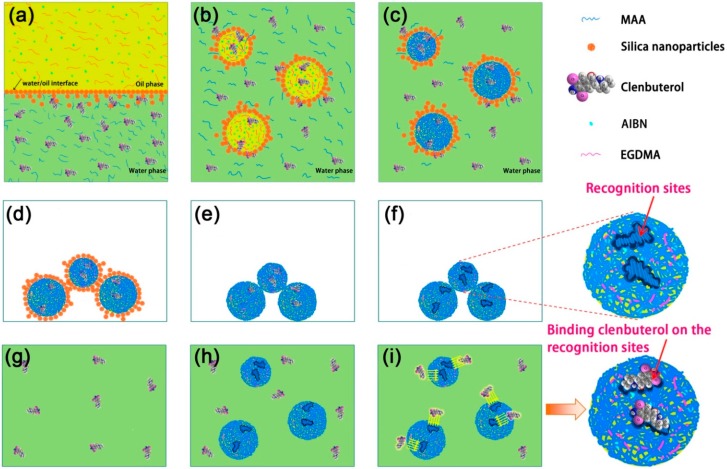
(**a**–**i**) Schematic illustration of the preparation of molecularly imprinted polymer microspheres (MIPMs) and their enrichment processing of clenbuterol. (**a**) Preparation of the water phase and oil phase containing the reactants needed for the Pickering emulsion polymerization. (**b**) Mixing the two phases to form oil-in-water droplets, where the oil droplets contained the cross-linking agent (i.e., EGDMA), initiator (i.e., AIBN) and part of clenbuterol molecules (as the molecular imprinting agent) and water phase was mainly comprised of the monomer (i.e., MAA); silica nanoparticles were dispersed along the interface between the oil phase and water phase. (**c**) Polymerization of the MAA to form the PMAA cross-linked by EGDMA. (**d**,**e**) Precipitation (**d**) and thorough washing (**e**) of the cross-linked PMAA microspheres. (**g**) The wastewater contaminated by clenbuterol. (**h**,**i**) Using MIPs to bind clenbuterol selectively (**i**).

The procedures for the synthesis of MIPMs are illustrated in Figure 1. Typically, the water phase was composed of the monomer, MAA (0.12 mL, 1.41 mmol), Triton X-100 (0.3%, 6 mL, 0.028 mmol), and silica nanoparticles (20 mg, 0.33 mmol), which was suffered to sonication treatment to disperse these silica nanoparticles thoroughly. Separately, the oil phase was prepared by mixing EGDMA (1.88 mL, 9.97 mmol), the template material (i.e., clenbuterol, 10 mg, 0.032 mmol), toluene (0.2 mL, 1.88 mmol) and the initiator (i.e., AIBN, 10 mg, 0.061 mmol) with the aid of sonication treatment for 10 min. The prepared water and oil phases were then mixed by intense agitation for 2 min, leading to the generation of the oil-in-water Pickering emulsion where the silica nanoparticles were dispersed along the interface between oil droplets and continuous water phase. The violent agitation at 70 °C allowed the initiation of the free-radical polymerization of the monomer (MAA) by AIBN in the oil droplets, and in the meanwhile, the cross-linking reaction also proceeded between the polymer chains and EGDMA. The polymerization and cross-linking reactions were performed for 16 h. The finally precipitated product was collected and dipped into a 30% HF solution for 12 h to etch away the silica nanoparticles on the surface of the polymer microspheres, which was then subjected to the Soxhlet solvent extraction with 50% methanol solution for 48 h. Such long extraction duration guaranteed that the template molecules (i.e., clenbuterol) were completely removed from the MIPMs, which could no longer be detected by electrospray ionization mass spectrometry (ESI-MS) and elemental analysis. The resulting polymer microspheres, with the average particle size of around 52 nm, were designated as the MIPMs. The non-molecularly imprinted counterpart (i.e., NIPMs), with the average particle size of about 42 nm, were also synthesized under the same preparation conditions except that no template was added during the Pickering emulsion polymerization. The mole ratio used for the preparation of the MIPMs was the optimized one. The insufficient monomer (MAA) did not give rise to the homogeneous polymer microspheres but bulk polymer, while excess MAA produced the microspheres showing nonspecific adsorption towards clenbuterol. Besides, insufficient crosslinker (EGDMA) could not result in a sufficient number of useful imprinting sites, whereas excess crosslinker caused the template molecules to be deeply embedded in the network and thus reduced the number of active imprinting sites.

### 2.3. Characterizations

The characterizations of the structural functionalities of the MIPM and NIPM samples were carried out by Fourier transform infrared (FTIR) spectroscopy and X-ray photoelectron spectroscopy (XPS) using an IRAffinity-1S spectrometer (Shimadzu Co., Kyoto, Japan) and XSAM800-XPS equipment (Kratos, UK), respectively. Scanning electron microscopy (SEM) images and elemental mapping images of the MIPMs and NIPMs were obtained using a HITACHI S-4800 Scanning Electron Microscope equipped with an energy dispersive X-ray (EDX) spectrometer (Quantax 70, Bruker Nano GmbH, Berlin, Germany). The Brunauer–Emmett–Teller (BET) measurements of the MIPMs and NIPMs were carried out with a Micromeritics ASAP 2020 nitrogen adsorption apparatus (Norcross, GA, USA). Before the BET test, the MIPMs and NIPMs samples were pre-degassed at 150 °C. The corresponding pore size distribution was determined by analyzing the desorption branch based on the Barrett–Joyner–Halenda (BJH) method. The mass spectrometric analysis was conducted using a 6540 UHD mass spectrometer (Agilent Technologies, Santa Clara, USA).

### 2.4. Adsorption Kinetics

To a glass vial, the MIPMs or the NIPMs (50 mg) were added, followed by the addition of a water solution of clenbuterol (10 mg/L, 10 mL). After the homogenization of the mixture imparted by shaking, the glass vial was equipped with a thermostatic water bath oscillator at an oscillation rate of 120 rpm for the adsorption processing. At different durations, the mixed solution was drawn for the measurements of the clenbuterol concentration in the solution, and the adsorbed amount was calculated on the basis of Equation (1) by LC-MS. The amount of the supernatant injected in the HPLC/MS system was 5 μL, and the test conditions were adopted as follows: electrospray ionization as the ion source, dry gas flow rate of 40 L/min, atomization pressure of 10 psi, and methanol as the mobile phase at the flow rate of 0.5 mL/min.

(1) $$qt=\left(\frac{C_{0}-C_{t}}{m}\right)\times V$$

where *C*_0_ and *C*_t_ represent the initial clenbuterol concentration and the concentration at time *t*, respectively.

The pseudo-first-order and pseudo-second-order kinetic models were adopted to analyze the adsorption kinetics according to Equations (2) and (3), respectively.

(2) $$q_{t}=q_{e}\left(1-e^{-k_{1}t}\right)$$

(3) $$q_{t}=\frac{q_{e}^{2}k_{2}t}{1+q_{e}k_{2}t}$$

where *q*_e_ represents the equilibrium adsorption amount (mg/g), *q*_t_ (mg/g) is adsorption amount at time *t*, and *k*_1_ and *k*_2_ designate the pseudo-first-order rate constant (1/min) and pseudo-second-order rate constant (g/mg min), respectively.

### 2.5. Adsorption Isotherm

To each of five glass vials, the MIPMs or the NIPMs (50 mg) were added. Then, the clenbuterol solutions at a volume of 10 mL, with different concentrations (10, 20, 30, 40, and 50 mg/L), were respectively charged into the five vials pre-loaded with polymer microspheres. After shaking for homogenization, these mixtures were placed into a thermostatic water bath oscillator, and the adsorption interactions proceeded at the oscillation rate of 120 rpm for 6.5 h which was sufficient for reaching equilibrium as proven by the test on the adsorption kinetics. Three different temperatures were considered, namely 30, 45 and 60 °C corresponding to the temperatures in Kelvin of 303, 318 and 333 K, respectively. After the equilibrium was reached, the resulting mixtures were centrifuged at 4000 rpm for 5 min before the measurement of the supernatants by LC-MS with the same test conditions as those described in the Section 2.4, and equilibrium adsorption amount (*q_e_*, mg/g) was calculated based on Equation (4).

(4) $$qe=\left(\frac{C_{0}-C_{e}}{m}\right)\times V$$

where *C*_0_ and *C*_e_ are the initial and equilibrium concentrations (mg/L) of clenbuterol, respectively, *m* represents the mass of the MIPM and NIPM adsorbents, and *V* is the volume (L) of the clenbuterol solution.

The commonly-used adsorption isotherm models were considered to analyze the present adsorption test results, in this case, Langmuir and Freundlich isothermal models that were described by Equations (5) and (6), respectively.

(5) $$q_{e}=\frac{q_{\max}k_{L}C_{e}}{1+k_{L}C_{e}}$$

(6) $$q_{e}=k_{F}C_{e}^{1/n}$$

where *q*_e_ and *C*_e_ are the equilibrium adsorption amount (mg/g) and clenbuterol concentration (mg/L), respectively, *k*_L_ and *k*_F_ are the Langmuir constant (L/g) and Freundlich constant (L/g), respectively, *q_max_* is the maximum adsorption capacity (mg/g), and 1/*n* is an empirical parameter related to adsorption intensity.

To assess whether the adsorption is favorable or unfavorable, Equation (7) can be referenced.

(7) $$R_{L}=\frac{1}{1+k_{L}C_{0}}$$

where *k*_L_ is the Langmuir constant (L/g), *C*_0_ presents the initial maximum clenbuterol concentration (mg/L), and *R*_L_ can be used to indicate the favorability of adsorption process, which are described below:

*R*_L_ > 1, unfavorable;
*R*_L_ < 1, favorable;
*R*_L_ = 1, Linear;
*R*_L_ = 0, irreversible.

### 2.6. Adsorption Thermodynamics

To study the inherent energy changes in the adsorption course, three basic thermodynamic parameters were measured, i.e., enthalpy change $\Delta H^{0}$, Gibbs free energy change $\Delta G^{0}$, and entropy change $\Delta S^{0}$. These thermodynamic parameters, $\Delta H^{0}$, $\Delta S^{0}$ and $\Delta G^{0}$ estimated for the adsorption of clenbuterol were described by Equations (8)–(10):

(8) $$\Delta G^{0}=\Delta H^{0}-T\Delta S^{0}$$

(9) $$\ln\left(k_{d}\right)=\frac{\Delta S^{0}}{R}-\frac{\Delta H^{0}}{RT}$$

(10) $$\log\left(\frac{q_{e}}{C_{e}}\right)=\frac{\Delta S^{0}}{2.303R}-\frac{\Delta H^{0}}{2.303RT}$$

where *C*_e_ is the equilibrium concentration of clenbuterol in solution (mg/L), *q*_e_ (mg/g) is the adsorbed amount at equilibrium. *T* represents the temperature in Kelvin (K), and *R* is the universal gas constant (8.314 J/(mol K)).

### 2.7. Competitive Adsorption Tests

A glass vial was loaded with the MIPMs or NIPMs (20 mg), and then a 10 mL mixed solution containing clenbuterol, terbutaline, salbutamol, and methyl red (each with a concentration of 1 mg/L) was poured into the glass vial pre-loaded with MIPMs or NIPMs. After homogenization imparted by shaking, the mixture was placed into a thermostatic water bath oscillator, and the adsorption interactions proceeded at the oscillation rate of 120 rpm for 6.5 h which was sufficiently long for making the adsorption system reach equilibrium. Then, the resulting mixture was centrifuged at 4000 rpm for 5 min, and the supernatant was drawn for the LC/MS measurement with the same test conditions as those described in the Section 2.4 and Section 2.5. The parameters related to the adsorption selectivity were calculated based on Equations (11)–(13) below.

(11) $$k_{d}=\frac{q_{e}}{C_{e}}$$

(12) $$k=\frac{k_{d}\left(\mathrm{clenbuterol}\right)}{k_{d}\left(x\right)}$$

(13) $$k^{\prime }=\frac{k\left(\mathrm{imprinted}\right)}{k\left(\mathrm{unprinted}\right)}$$

where *q*_e_ is the equilibrium adsorption amount (mg/g), *C*_e_ is the equilibrium concentration of the adsorbate in the solution (mg/L), *k*_d_ represents the distribution coefficient (L/g), *k* is the selectivity coefficient, *x* represents competing species against clenbuterol, including terbutaline, salbutamol, and methyl red, and *k′* is the relative selectivity coefficient.

### 2.8. Impact of the Solution as the Medium for the Adsorption of Clenbuterol onto MIPs and NIPMs

Various media were employed to prepare 10 mg/L clenbuterol solutions, including water, a water solution of ammonium acetate (4.6 mmol/L), a water solution of 0.3%TX-100 (4.6 mmol/L), acetonitrile, and an acetonitrile solution of ammonium acetate (4.6 mmol/L). These prepared solutions were separately added into the vial pre-loaded with 50 mg MIPMs or NIPMs. After the mixtures had been homogenized by shaking, they were placed in a thermostatic water bath oscillator with an oscillation rate of 120 rpm/min for the isothermal adsorption for 5 h. The processed clenbuterol solutions were centrifuged for 5 min, and the supernatants were withdrawn for the LC-MS measurement. Based on the equilibrium adsorption quantity calculated, the imprinting factor (IF) was estimated according to the following Equation (14).

(14) $$\mathrm{IF}=\frac{q_{e}\;\left(MIPMs\right)}{q_{e}\;\left(NIPMs\right)}$$

### 2.9. Reliability of the Method Used for the Fabrication of MIPMs

A new batch of the clenbuterol-imprinted polymer microspheres was prepared to prove that the present method for the fabrication of MIPMs was reliable, and the newly prepared sample was designated as MIPMs-2. The adsorption isotherm was then measured for MIPMs-2 according to the isothermal adsorption test on MIPMs. Through comparison of the parameters obtained by fitting the adsorption isotherms for the adsorption systems with MIPMs and MIPMs-2, the reliabilities of the present fabrication method and isothermal adsorption test could be assessed.

### 2.10. Test on the Recyclability of the MIPMs

The prepared MIPMs were used as the stationary phase of the molecularly imprinted solid-phase extraction (MISPE) column. Briefly, the deionized water and methanol were firstly used to thoroughly wash the MISPE column walls which were then dried in air. Sieve plates were employed to seal the open ends of the MISPE column, and thus the falling of the MIPMs was prevented. The filled MISPE column was further washed with a mixture of methanol and acetic acid (9/1, *v*/*v*) at a volume of 10 mL and then with 2 mL methanol. A water solution of clenbuterol (10 μg/L, 100 mL) subsequently passed through the washed MISPE column at a flow rate of about 5 mL/min under negative pressure. Finally, the MISPE column adsorbed with clenbuterol was successively eluted with a mixture of methanol/acetic acid (9/1, *v*/*v*) at volumes of 1 mL, 2 mL, and 2 mL. After the residual solution had been drained out of the MISPE column, the eluted solution was examined using LC-MS based on the test conditions as mentioned earlier. The MISPE test was repeated nine times, and the spike recovery was calculated for each cycle. Consequently, the recycling performance of the MISPE column with the MIPMs was evaluated based on the comparison of the spike recoveries calculated.

## 3. Results and Discussion

Figure 1 depicts the main content of this study, that is, investigation of the MIPMs for the enrichment of clenbuterol. More specifically, through the Pickering emulsion polymerization and molecular imprinting technique, the MIPMs were fabricated. The comparison between the prepared MIPMs and NIPMs allowed the clarification of the impact of the molecular imprinting on the polymer microspheres synthesized by Pickering emulsion polymerization. We found that the molecular imprinting could not only change the size of these polymer microspheres but alter their surface functionalities. The molecular imprinting should possess a unique capability to leave recognition cavities as depicted in Figure 1 and evidenced by the BET measurements. The N_2_ adsorption-desorption isotherms and BJH pore size distribution plots are provided in Figures S1 and S2 in the Electronic Supporting Information (ESI), respectively. The results obtained via BET measurements are summarized in Table 1. The MIPMs showed a significantly smaller average pore width that was estimated to be around 6 nm relative to that of NIPMs (more than 150 nm), as shown in Figure S2 in the ESI. Notably increased cumulative pore volume and specific surface area can also be measured for MIPMs as compared to NIPMs (Table 1). For NIPMs, the type II isotherm can be assigned (Figure S1 in the ESI), indicating that NIPMs belong to a non-porous or macroporous material [49], and we hypothesized that the microsphere accumulation could generate gaps among these microspheres, which were tested as the macropores in the N_2_ sorption test. By contrast, the N_2_ adsorption onto MIPMs results in the formation of the type IV isotherm, which suggests the existence of mesoporous structures due to the generated recognition sites. The non-closure of the hysteresis loop implies incomplete removal of adsorbate from narrow pores [49]. The comparison of the N_2_ sorption measurement results between MIPMs and NIPMs revealed the success in the manufacturing of the polymer microspheres via clenbuterol imprinting which left recognition cavities complementary to the size of clenbuterol molecules. Solvent extraction processing was most likely to loosen the structure of MIPMs to a certain extent, resulting in improved specific surface area and pore volume. These beneficial results imparted via clenbuterol imprinting endow the MIPMs with great potential for the clenbuterol enrichment applications.

**Figure 2:**
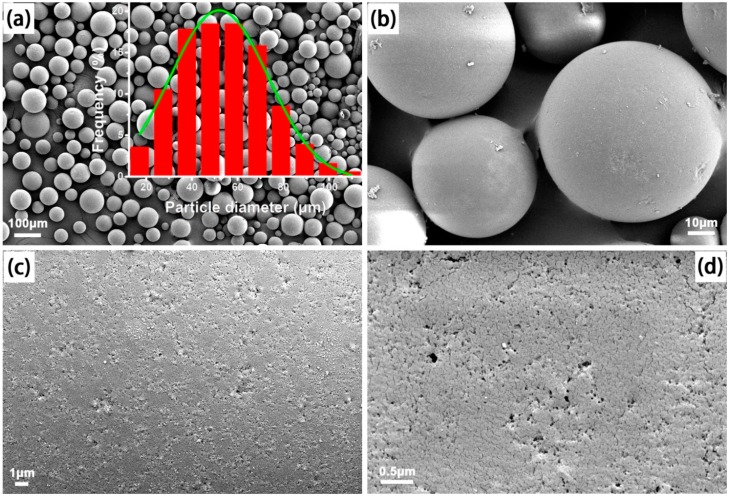
(**a**–**d**) SEM images of the MIPMs with the molecular imprinting treatment and the inset in (**a**) shows the particle size distribution histogram.

**Figure 3:**
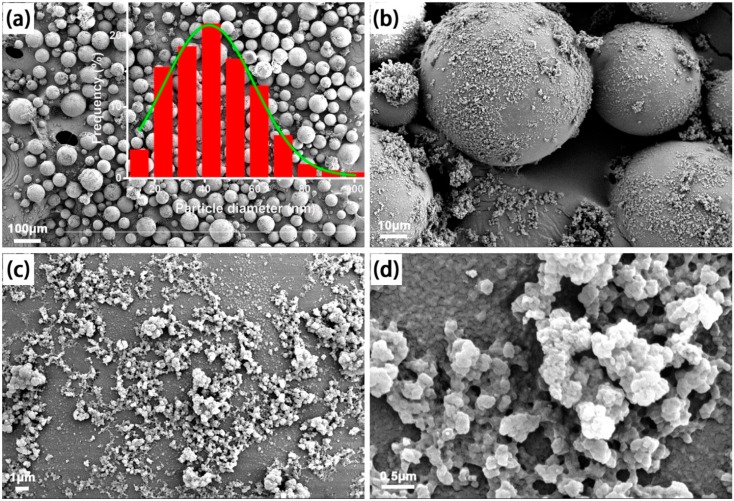
(**a**–**d**) SEM images of the NIPMs prepared without the molecular imprinting treatment and the inset in (**a**) shows the particle size distribution histogram.

**Figure 4:**
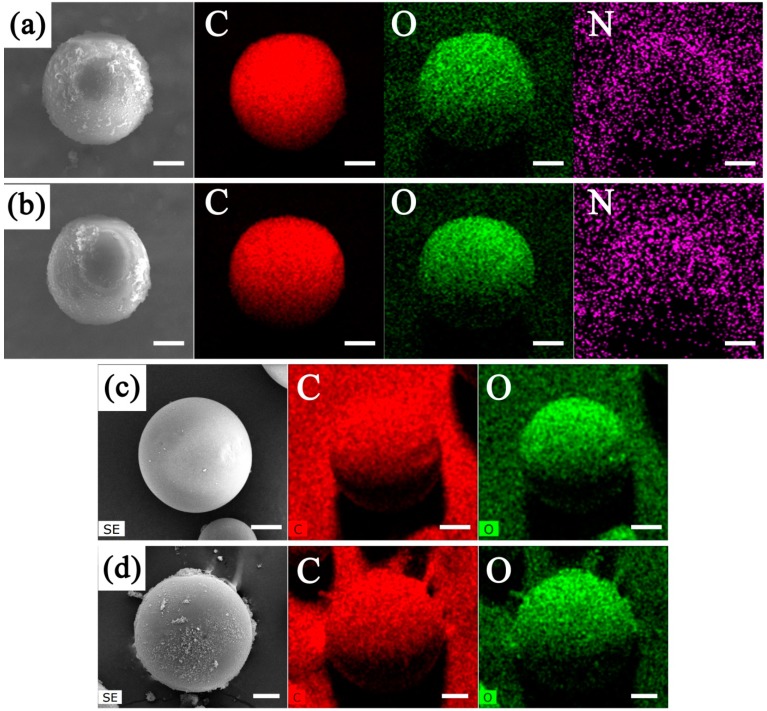
(**a**–**d**) Elemental mapping images of the intermediate polymer microspheres prepared before the procedure of the etching treatment with HF (**a**), the intermediate polymer microspheres prepared before the procedure of the Soxhlet extraction treatment (**b**), MIPMs (**c**) and non-molecularly imprinted polymer microspheres (NIPMs) (**d**), together with the corresponding SEM images presented on the leftmost side; the scale bars in all of the images are 20 μm.

From the SEM images of the MIPM and NIPM samples presented in Figure 2 and Figure 3, respectively, notable differences can be observed. The average particle size of MIPMs was estimated to be approximately 52 nm, larger than that of NIPMs (around 42 nm). The particle size distribution histograms are provided in the insets of Figure 2 and Figure 3. Without the molecular imprinting, many tiny particles were noted on the NIPM surface, in stark contrast to the rather clean surface of the MIPMs. These results imply that the addition of the template molecules (i.e., clenbuterol) could improve the Pickering emulsion polymerization quality, most likely resulting from the clenbuterol-assisted promotion of the emulsion stability. Apart from the molecular imprinting template, clenbuterol might also serve as a surfactant, in that a hydrophobic moiety (–C(CH_3_)) at one end and a hydrophilic moiety (–NH_2_) at the other exist in the molecular structure of clenbuterol (Figure S3 in the ESI). Such dual roles of the surfactant and molecular imprinting template played by clenbuterol can impart an advantage of the present strategy for the fabrication of functional polymeric materials for processing clenbuterol. Importantly, small microcracks and micropores could be found on the surface of MIPMs (Figure 2c,d), in contrast to the more seamless surface of NIPMs (Figure 3c), which was an indication of the useful molecular imprinting generated on the polymer microspheres. These findings are also in good consistency with the results obtained via N_2_ sorption measurements. These generated microcracks and micropores would help to the selective enrichment of clenbuterol due to the molecular imprinting effect. Elemental mapping images presented in Figure 4a–d confirmed that only C and O elements could be detected in both the MIPMs (Figure 4c) and NIPMs (Figure 4d), indicating that the template molecules were extracted entirely out of the MIPMs since the N element exists in the template (Figure S3 in the ESI). We also measured the elemental mapping images of the intermediate samples before the procedure of etching treatment with HF and the procedure of the Soxhlet extraction treatment, which are shown in Figure 4a,b, respectively. For both the intermediate products, the N element could be probed, revealing that the successful incorporation of clenbuterol molecules (containing the N element) into the polymer microspheres. Even though the HF is a strong etching agent, it could not exert a significant impact on the clenbuterol template located in the polymer microspheres, as the N element was clearly presented in Figure 4b. Therefore, it is necessary to further extract the clenbuterol template out of the polymer microspheres by an approach following the etching treatment with HF. To this end, we adopted the Soxhlet extraction by considering its ease in operation, albeit with a long extraction duration. After the Soxhlet extraction, the N element could no longer be detected (Figure 4c), confirming that the Soxhlet extraction was powerful for the entire extraction of the clenbuterol template out of the cross-linked polymer microspheres.

**Figure 5:**
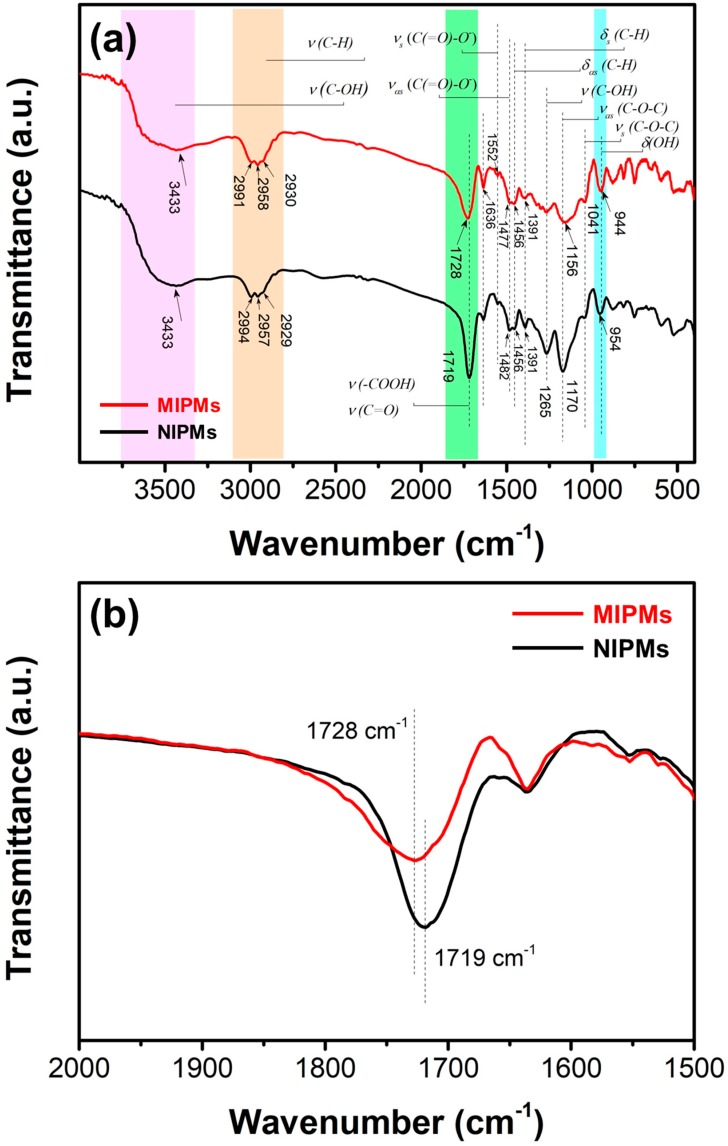
(**a**,**b**) FTIR spectra of the prepared NIPMs and MIPMs samples; the magnified FTIR spectra in the selected wavenumber range of 2000–1500 cm^−1^.

Functional groups on the MIPMs and NIPMs were then probed by FTIR spectroscopy, and the results are presented in Figure 5a,b. We marked most of the characteristic FTIR absorption peaks and their corresponding functionalities in Figure 5. For the MIPMs, the presence of the carboxyl groups of the poly(methacrylic acid) (PMAA) moiety was most clearly reflected by the FTIR absorption around 3433 and 1728 cm^−1^ which were indexed to the stretching vibrations of –OH and –COOH, respectively. The FTIR absorptions revealed the methyl, methylene, and methane in the MIPMs at approximately 2991, 2958, 2930, 1456 and 1391 cm^−1^ which were assigned to the stretching and bending vibrations of the C–H group. We also noted the presence of carboxylate functionalities at 1522 and 1477 cm^−1^ corresponding to the symmetric and asymmetric stretching vibrations of carboxylate as formed by the partial ionization of carboxyl groups. In comparison between the MIPMs and NIPMs, the apparent difference in the characteristic FTIR absorption was observed due to the stretching vibration of carboxyl groups, as highlighted in Figure 5b. For the MIPMs, this absorption was detected at about 1728 cm^−1^, which was shifted to approximately 1719 cm^−1^ as for the NIPMs. Such a kind of redshift has been elucidated to be the enhancement of hydrogen bonding interactions [50]; in other words, molecular imprinting treatment caused the breakage of some hydrogen bonding between PMAA chains such as COOH…HOOC. This weakened hydrogen bonding interactions might also be a result of a decrease in the number of carboxyl groups. The molecular imprinting not only created cavities with the size matched with the clenbuterol molecule but also modulated the surrounding chemical properties, and in this study, the molecular imprinting was demonstrated to change the oxygen functionalities and their hydrogen-bonding interactions. The weakened hydrogen bonding is favorable for the adsorption applications since less diffusion resistance to the adsorbate molecules would be generated by the hydrogen bonding.

**Figure 6:**
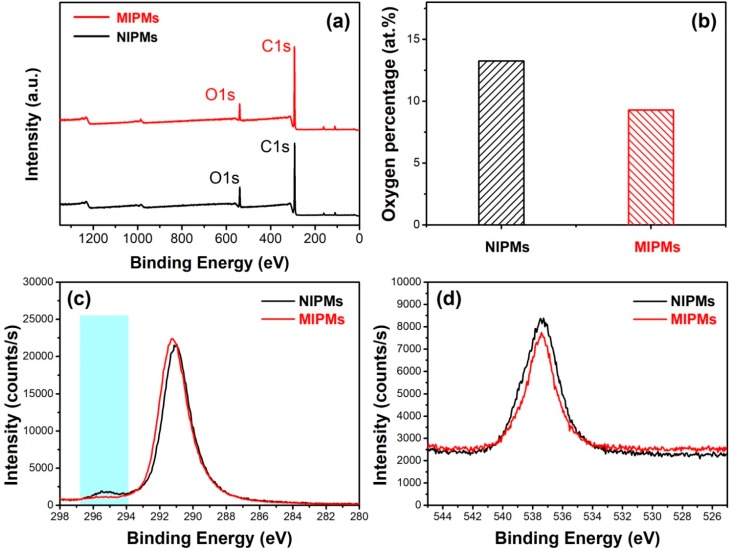
(**a**–**d**) XPS spectra of the prepared NIPMs and MIPMs samples: (**a**) XPS survey spectra, (**b**) comparison on the oxygen atomic percentage based on the XPS survey spectra between the NIPM and MIPM samples, and (**c**,**d**) high-resolution XPS C1s (**c**) and O1s (**d**) core-level spectra.

XPS technique was further employed to consolidate the above analysis based on the FTIR data, and the results are provided in Figure 6a–d and Table 2. From the XPS survey spectra presented in Figure 6a, the atomic percentage of oxygen was estimated, and the results were exhibited by a comparison histogram shown in Figure 6b. The oxygen content was lowered for MIPMs relative to NIPMs, which thereby elucidates that the weakened hydrogen bonding as proven by FTIR spectroscopy was attributed to the decease of oxygen-containing functional groups. High-resolution XPS C1s and O1s core-level spectra are further provided in Figure 6c,d, respectively. The peak around 295 eV assigned to the carboxyl group was significantly weakened for the MIPMs relative to the NIPMs (as highlighted by light blue shading in Figure 6c), which thus suggested that the decrease of the carboxyl groups at least partially caused the lowered oxygen atomic content. The lowered concentration of the oxygen-containing functional groups was also unambiguously ascertained by the XPS O1s spectra (Figure 6d) since the intensity of the primary peak on the O1s spectrum was decreased for the MIPMs as compared to that for the NIPMs. An appropriate decrease in the oxygen group concentration is necessary to impart the MIPMs with high specificity by considering that most of the oxygen-containing functional groups interact with the organic species in a nonspecific way, e.g., through hydrogen bonding interactions [51]. The clenbuterol molecular imprinting led to the generation of the binding sites with an appropriate size and surface properties, facilitating the highly selective enrichment of clenbuterol.

**Figure 7:**
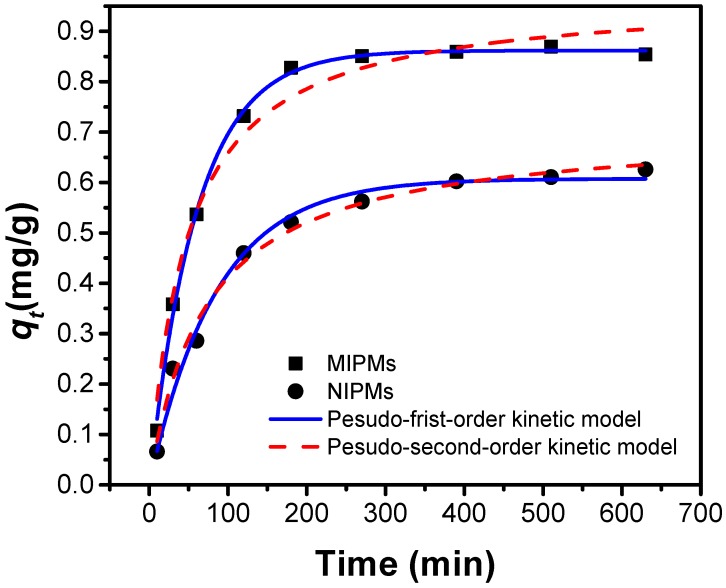
Adsorption kinetics for the uptake of clenbuterol onto the NIPM and MIPM adsorbents, together with the data fitting based on pseudo-first-order and pseudo-second-order kinetic models.

**Figure 8:**
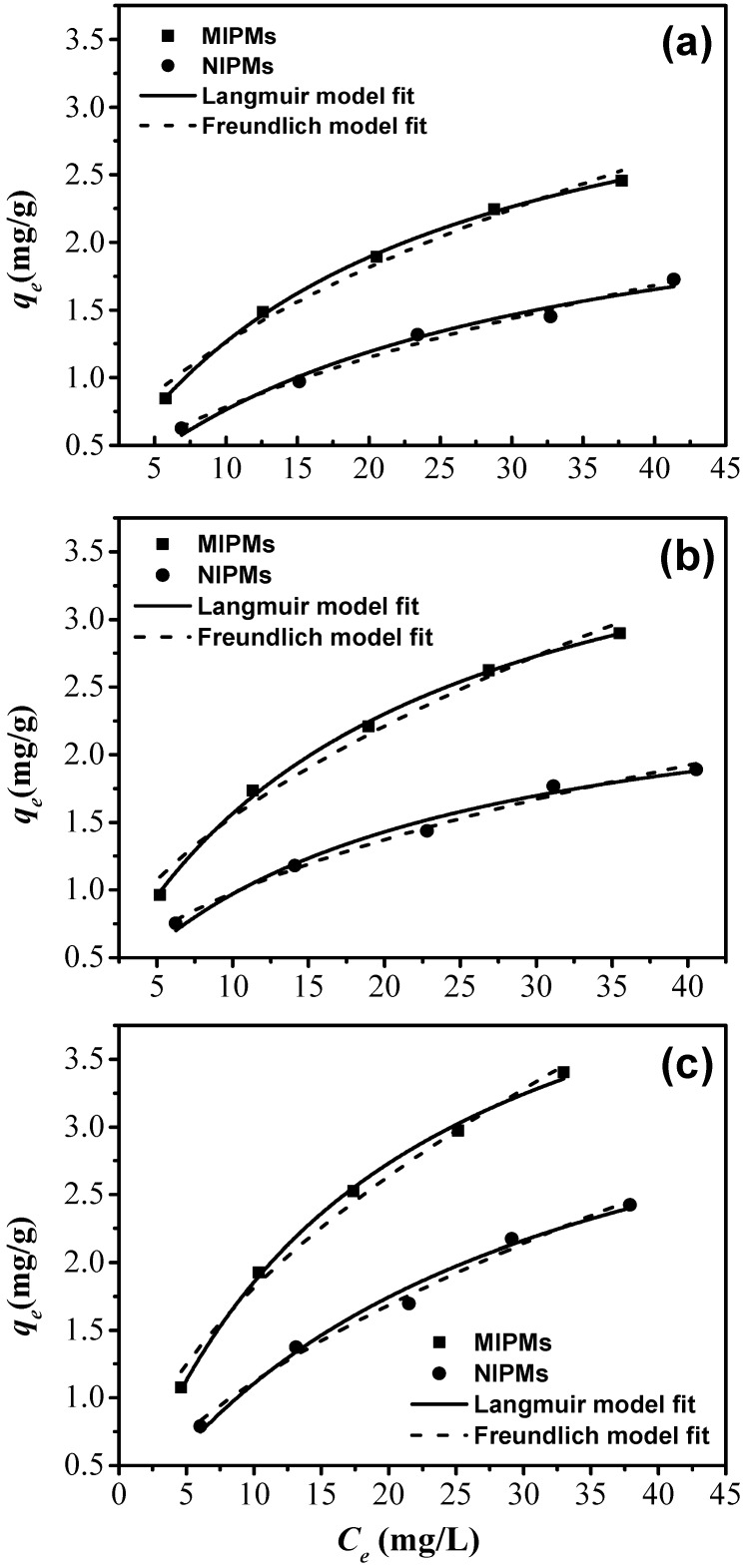
(**a**–**c**) Isothermal analysis for the enrichments of clenbuterol onto the NIPMs and MIPMs at 30 °C (**a**), 45 °C (**b**), 60 °C (**c**).

**Figure 9:**
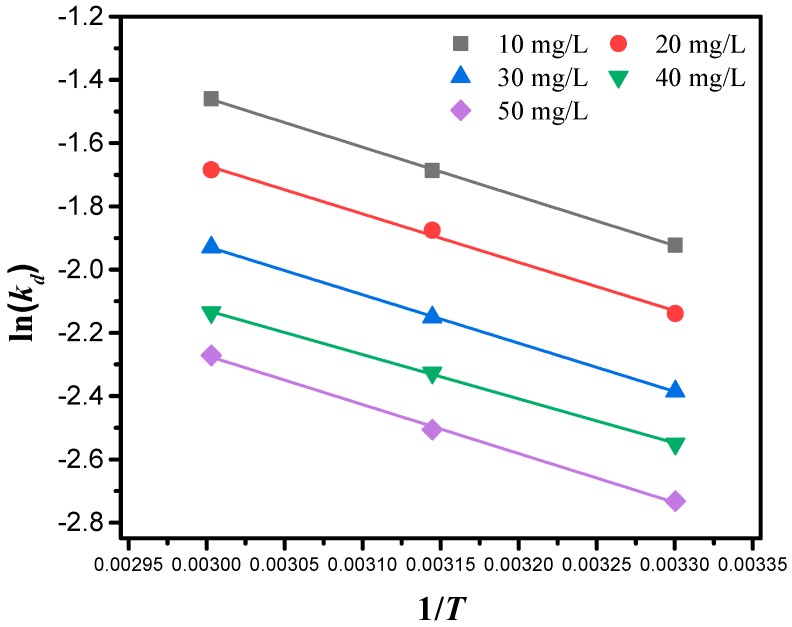
Plots of ln(*k*_d_) as a function of 1/*T* for different MIPMs-based adsorption systems with a series of initial clenbuterol concentrations.

**Figure 10:**
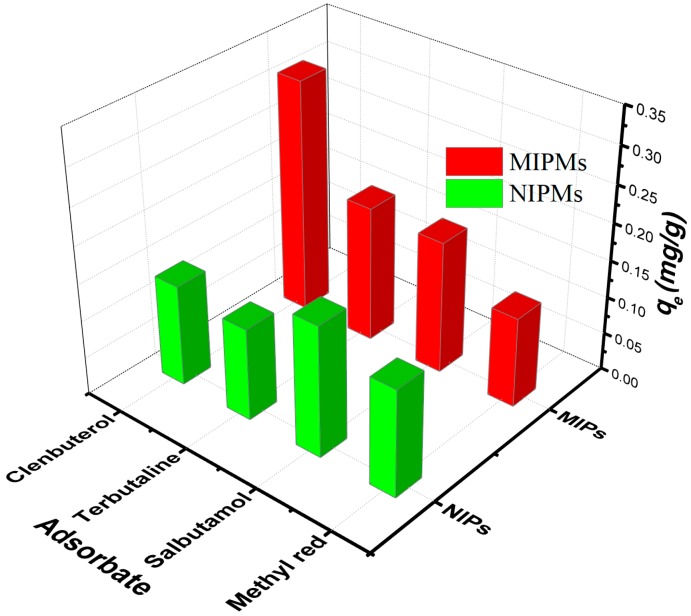
Selective separation of clenbuterol by the MIPMs and NIPMs from a mixture containing different competing organic species with high structural similarities to clenbuterol, including terbutaline, salbutamol, and methyl red.

All of the results confirmed the successful preparation of polymer microspheres via Pickering emulsion polymerization and effective molecular imprinting of the as-prepared polymer microspheres, with binding sites created for the enrichment of clenbuterol. The adsorption performance of the MIPMs was then systematically studied and compared with that of the NIPMs, and the results are presented in Figure 7, Figure 8, Figure 9 and Figure 10, and Tables S1–S3 in the ESI. The adsorption kinetics was firstly investigated (Figure 7 and Table S1 in the ESI). The adsorption of clenbuterol onto both the MIPMs and NIPMs was fast at the beginning 2.5 h and gradually became slow until the equilibrium was reached. There were abundant binding sites unoccupied by the clenbuterol molecules at the initial stage, and when clenbuterol molecules gradually occupied the binding sites, the adsorption showed down. Besides, MIPMs better followed the pseudo-first-order kinetic model relative to the pseudo-second-order kinetic model, which was opposite to the adsorption kinetics in the case of the NIPMs as the adsorbent that better obeyed the pseudo-second-order kinetic model, as judged by the correlation efficient (*R*^2^). Such a discrepancy between the adsorption kinetics as for the adsorption systems with the MIPM and NIPMs was assumed to stem from the reduced content of oxygen-containing functional groups in the MIPMs relative to the NIPMs, which weakened the chemical interactions between oxygen groups (especially carboxyl groups, as evidenced by Figure 5 and Figure 6) of the adsorbent and clenbuterol via electron sharing, transferring and exchanging. The better fitting to the pseudo-second-order kinetic model has been presumed to be probably originated from the chemisorption [52]. The reduction of the oxygen functional groups on the MIPMs weakened the chemical interactions between the MIPMs and clenbuterol, which enabled the change of the adsorption kinetics from the pseudo-second-order kinetic model (for NIPMs) to the pseudo-first-order kinetic model (for MIPMs). Nevertheless, the adsorption capacity of the MIPMs was overwhelmingly more significant than that of NIPMs (Figure 7), probably resulting from the generation of many molecular imprinting sites complementary to clenbuterol in the size and surface properties. These created sufficient imprinting sites would endow the MIPMs with high binding specificity to the clenbuterol molecules, together with a higher enrichment capacity as compared to the NIPMs, albeit with reduced oxygen-containing functional groups.

The isothermal adsorption results are provided in Figure 8, and Table S2 in the ESI and three different temperatures including 30, 45, 60 °C were investigated, with the corresponding results provided in Figure 8a–c, respectively. For the adsorption system with the MIPMs at all the investigated temperatures, the isothermal adsorptions were better followed by the Langmuir isotherm relative to that by the Freundlich isotherm. The Langmuir model assumes that the adsorption sites on the MIPMs are homogeneous and energetically equivalent and that the adsorption proceeds at specific homogeneous sites within the MIPMs [52]. We can thus speculate that the molecularly imprinted sites are homogeneously distributed over the MIPMs, and these sites are energetically equivalent. Additionally, the Langmuir model also indicates the monolayer coverage of clenbuterol onto the MIPMs [53]. Only are the recognition sites not occupied by the clenbuterol molecules; they are available for the adsorption. The results can also reflect that molecular imprinting treatment enables the MIPMs to be more homogenous in the structure by reducing the heterogeneous sites on the MIPMs such as carboxyl groups, as evidenced by the XPS spectra. To examine the reliability of the present method for the preparation of MIPMs, we also prepared the second batch of MIPMs, namely MIPMs-2, for which the adsorption isotherm was also tested, as shown in Figure S4 and Table S3 in the ESI. As expected, the adsorption of clenbuterol on the MIPMs-2 sample also resulted in the adsorption isotherm, which also exhibited the better fitting to the Langmuir model as compared to the Freundlich model. This result thus elucidates the reliability of the present fabrication method for obtaining similar MIPMs from different batches. 

On the contrary, the isothermal adsorption of clenbuterol onto the NIPMs better obeyed the Freundlich isotherm in comparison with the Langmuir isotherm. The Freundlich equation is an empirical equation adopted to describe heterogeneous systems, and it is not restricted to the monolayer adsorption [52]. The more abundant oxygen groups on NIPMs (relative to those on MIPMs) make the oxygen groups more likely to be heterogeneously distributed over the NIPMs. The more significant number of oxygen groups make them more possible to exist in different types, with different binding affinities, on the NIPMs. The heterogeneous distribution of the different types of oxygen groups on the NIPMs enables the adsorption isotherm to follow the Freundlich equation.

The difference in the concentration of oxygen groups and the intensity of hydrogen bonding interactions caused a discrepancy between the MIPMs and NIPMs in both the adsorption kinetics and isotherms. The stronger hydrogen bonding interactions between the surface oxygen groups might deactivate their adsorption activity towards clenbuterol molecules which could be more favorably adsorbed onto the sites free from the hydrogen bonding interactions with other oxygen groups. It was also noted that both the adsorption systems with the MIPMs and NIPMs exhibited promotion of the maximum adsorption capacity with increasing the temperature, implying both endothermic adsorption systems. From the thermodynamic analysis results provided in Figure 9 and Table S4 in the ESI, significant parameters including Δ*H*°, Δ*S*°, and Δ*G*° were calculated for the adsorption of clenbuterol onto the MIPMs. The positive Δ*H*° and Δ*S*° values indicate the endothermic nature and randomness of the adsorption of clenbuterol onto the MIPMs [54]. The positive Δ*G*° value reveals that the adsorption reaction requires energy. Since a decrease of Δ*G*° could increase the reaction rate, higher temperatures were beneficial to the adsorption in this case, in consistence with the aforementioned endothermic adsorption process.

We also investigated the influence of the type of solution used as the medium for the adsorption experiment, and the results are presented in Figure S5 and Table S5 in the ESI. It can be noted that MIPMs exhibited a higher adsorption capacity toward clenbuterol in comparison with NIPMs, irrespective of the media employed. Additionally, a higher capacity of the adsorption of clenbuterol was achieved in the water-based media (including pure water, a water solution of ammonium acetate and a water solution of Triton X-100), in comparison to that in the acetonitrile-based media (including pure acetonitrile and an acetonitrile solution of ammonium acetate). Nevertheless, the higher selectivity was obtained in the acetonitrile-based media, as evidenced by the much-improved imprinting factor for the adsorption system with the acetonitrile-based media relative to the water-based media. These results thus reveal that, in addition to the structure and properties of the MIPMs, the medium used for the adsorption experiment also plays a significant role in deciding the final adsorption performance, including adsorption capacity and selectivity.

The above adsorption studies clarified that the MIPMs exhibited an improved adsorption capacity relative to the NIPMs, indicative of the useful molecular imprinting effect on the polymer microspheres. The promoter for this improvement was the recognition sites as generated by the effective molecular imprinting treatment of the polymer microspheres. Whether these recognition sites showed selectivity to clenbuterol was the next that we would be demonstrated, and the results are shown in Figure 10, and Figure S6 and Table S6 in the ESI. The MIPMs showed much larger selectivity coefficient (*k*) values in comparison with those of the NIPMs, together with the relative selectivity coefficient (*k′*) values largely exceeding 1. This result is an indication that, in comparison with the NIPMs, the MIPMs exhibited an enhanced selectivity to clenbuterol among potential competing analogs with high structural similarities (Figure S1 in the ESI). The specific EIS-MS spectra of the mixed analyte solution with clenbuterol, methylene blue, terbutaline, and Salbutamol before and after processing with NIPMs or with MIPMs are presented in Figure S6 in the ESI.

**Figure 11:**
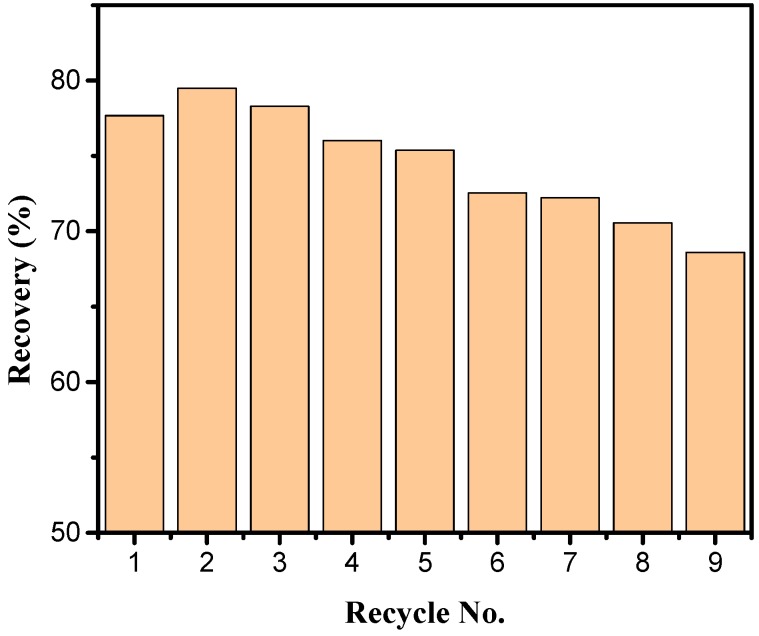
Recycling performance of the MIPMs when used as the stationary phase of the MISPE column for processing of clenbuterol solution through the evaluation of the spike recovery for each cycle.

We also fabricated a MISPE column using the MIPMs as the stationary phase, and the spike recovery was calculated. A total of 9 cycles were examined, and the spike recovery calculated for each cycle was compared, with the result provided in Figure 11. The results show that the MISPE column with the MIPMs can be reused for many cycles without significant degradation of the MISPE column performance. The slight decrease in the spike recovery might be caused by the somewhat contamination of the imprinting sites on the MIPMs during the repeated usage of the MISPE column equipped with the MIPMs.

## 4. Conclusions

We have effectively employed Pickering emulsion polymerization and molecular imprinting manufacturing to produce the MIPMs with recognition sites complementary to the clenbuterol template in the size and functionalities. Numerous cavities are generated on the surface of MIPMs as a result of molecular imprinting, in stark contrast to the NIPM surface. On the other hand, relative to the clean MIPM surface, many tiny particles are observed on the NIPM surface, which indicates that the presence of the clenbuterol template during the Pickering emulsion polymerization helps to enhance the polymerization quality attributed to the clenbuterol-aided stabilization of the emulsion. Besides, the existence of the clenbuterol template also enables the average particle size of the resulting MIPMs to be increased as compared to that of the NIPMs; the clenbuterol template can help to stabilize the oil droplets, even in larger sizes in the continuous water phase. Interestingly, molecular imprinting also causes a reduction of the oxygen-containing functional groups (such as carboxyl groups) on the MIPMs, relative to those on the NIPMs, which is beneficial for the selective uptake of the clenbuterol in the subsequent adsorption tests since too many oxygen groups will normally debase the specificity. The MIPMs show much better adsorption performance towards clenbuterol in comparison with the NIPMs, and more importantly, they also exhibit an excellent selectivity to clenbuterol among some potential analogs with high structural similarities. The recyclability of the MIPMs in the clenbuterol enrichment is also manifested through running the MISPE column equipped with the MIPMs nine times. Furthermore, the reliability of the fabrication method is also verified by the comparison of two batches of the MIPMs. Therefore, these MIPMs possess great potential for environmental monitoring and purification applications towards clenbuterol and other threatening species posing a severe threat to human life safety.

## Figures and Tables

**Table 1:** Summary of the critical information obtained from the Brunauer–Emmett–Teller (BET) measurements, including BET surface area, the volume of pores, and average pore width.

Sample	BET Surface Area	Volume of Pores	Average Pore Width
	m²/g	cm³/g	nm
MIPMs	14.2265	0.029734	5.7847
NIPMs	3.7190	0.008051	151.0666

**Table 2:** Summary of the information obtained for NIPMs and MIPMs based on the corresponding XPS survey spectra, including peak position, full width at half maximum (FWHM), peak area, and atomic percentages of carbon and oxygen.

	Name	Peak BE	FWHM	Area (P)	Atomic
		eV	eV	CPS.eV	%
NIPMs	O1s	539.37	3.3	272,588.87	13.25
	C1s	292.06	2.77	739,143.34	86.75
MIPMs	O1s	539.98	2.91	194,760.04	9.29
	C1s	292.88	2.58	787,419.49	90.71

## Supplementary Materials

The following are available online at https://www.mdpi.com/2073-4360/11/10/1635/s1.
