# Supplementary material for: Selective Enrichment of Clenbuterol onto Molecularly Imprinted Polymer Microspheres with Tailor-made Structure and Oxygen Functionalities
Source: Polymers (Basel). 2019 Oct 10;11(10):1635. doi: 10.3390/polym11101635 (PMC6835586; doi:10.3390/polym11101635)
Supplement: Supplementary file 1 [file polymers-11-01635-s001.pdf]

Electronic Supporting Information
Selective Enrichment of Clenbuterol onto Molecularly Imprinted Polymer Microspheres with Tailor-made Structure and Oxygen Functionalities

Xiangyun Zhao[1], Yuliang Mai*[,1], Dongchu Chen[2], Min Zhang[2] and Huawen Hu*[,2]

[1] Guangdong Provincial Key Laboratory of Industrial Surfactant, Guangdong Research Institute of Petrochemical and Fine Chemical Engineering, Guangzhou 510006, P. R. China
[2] School of Materials Science and Energy Engineering, Foshan University, Foshan, Guangdong 528000, PR. China

TOC figure

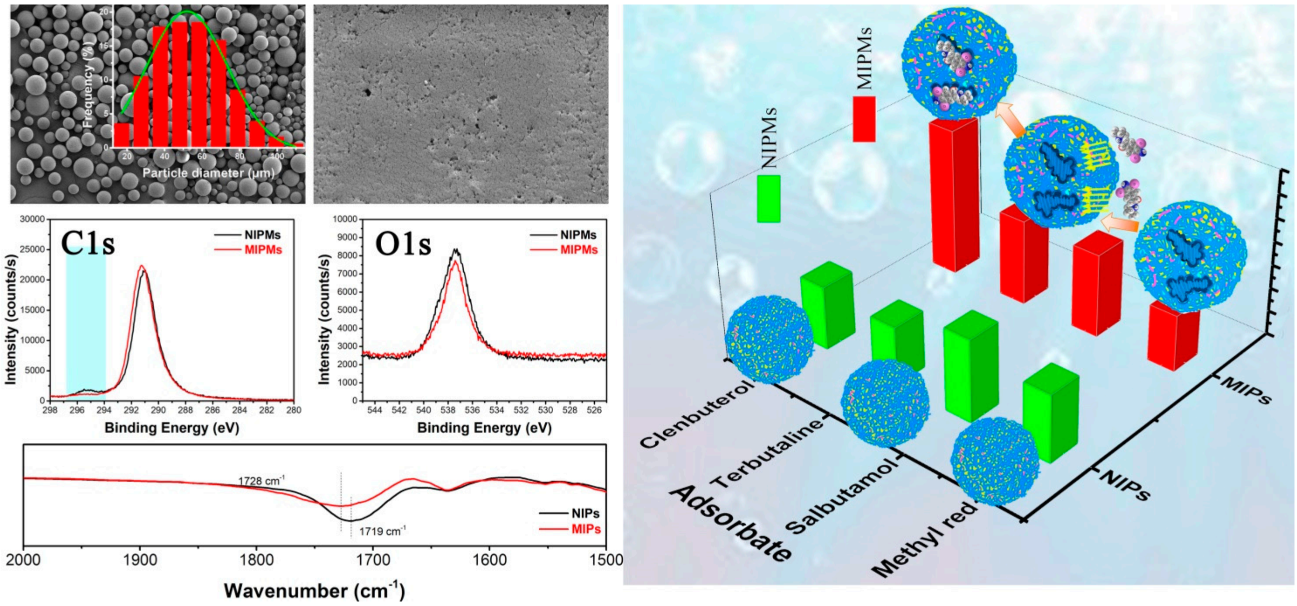

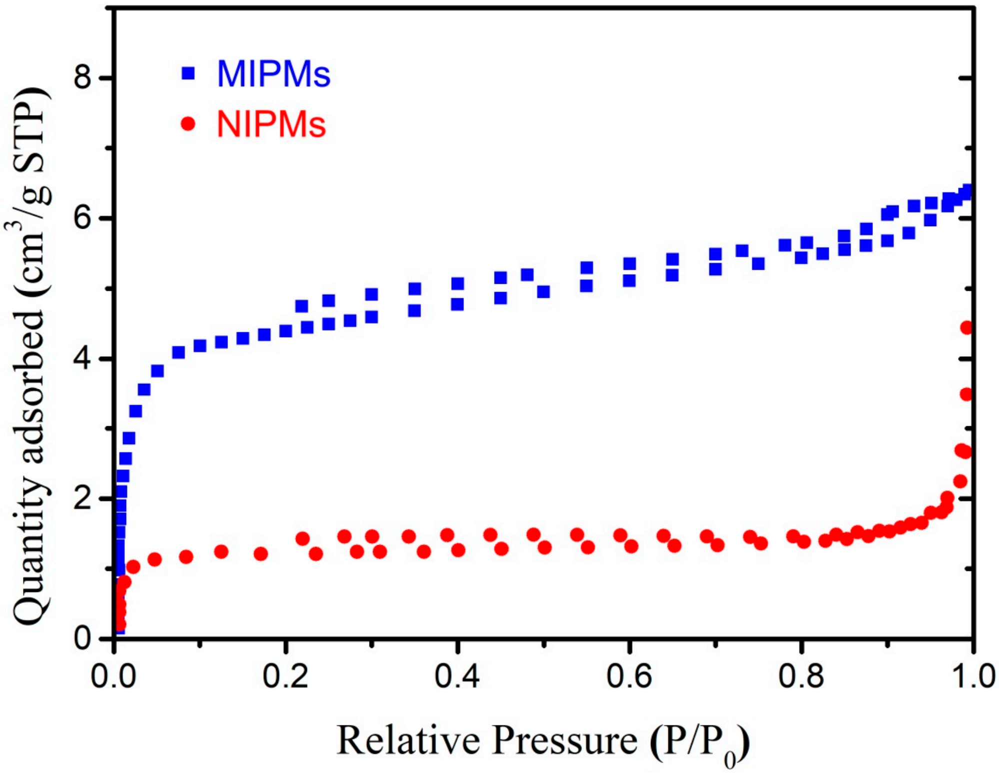

Figure S1. N_2 adsorption-desorption isotherms measured for MIPMs and NIPMs.

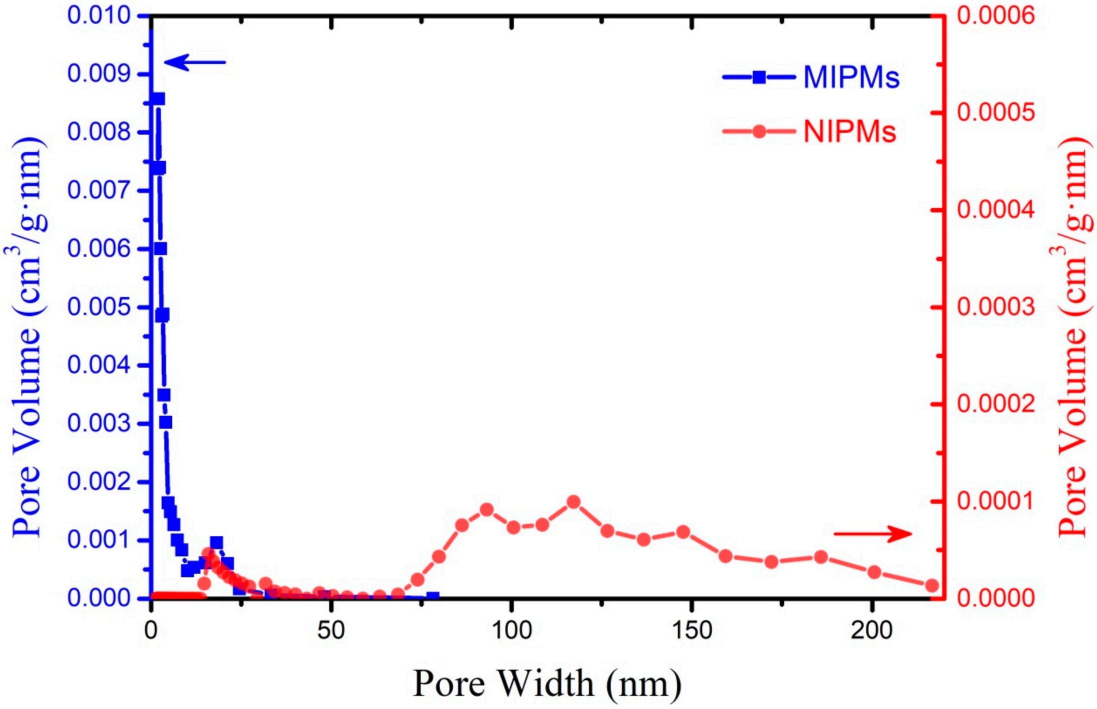

Figure S2. Barrett–Joyner–Halenda (BJH) pore size distribution plots of MIPMs and NIPMs derived from the desorption branches.

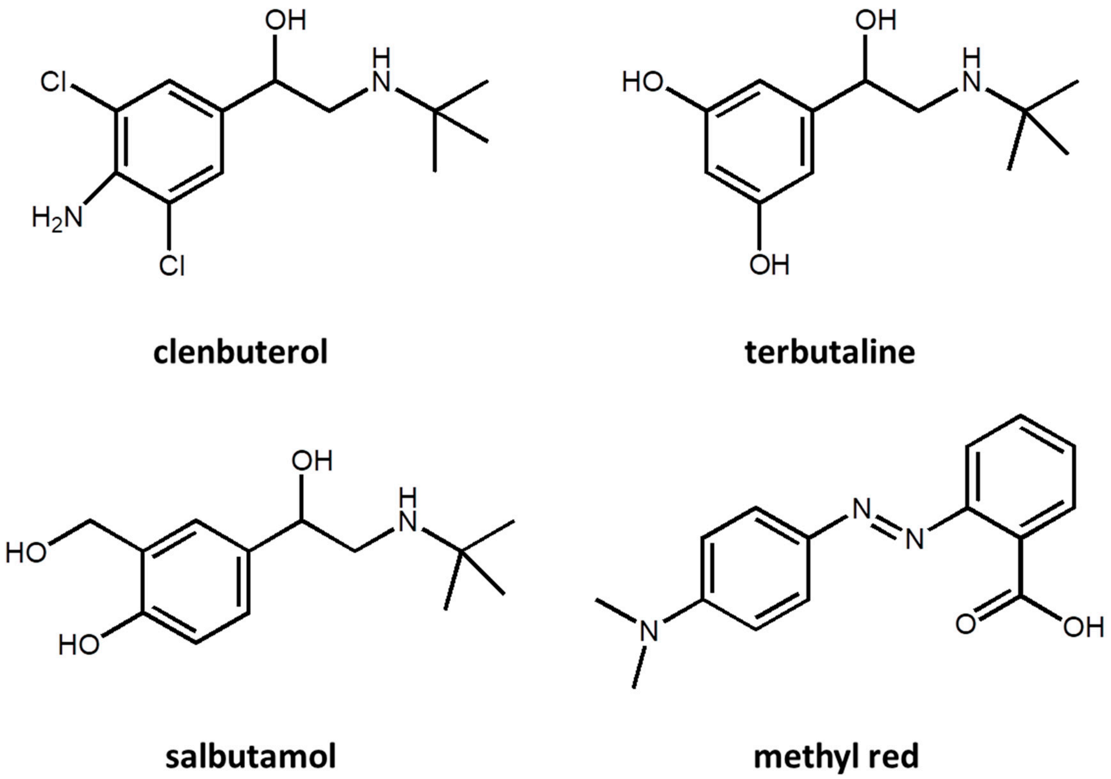

Figure S3. Molecular structures of clenbuterol and its competing species with structural similarities, including terbutaline, salbutamol, and methyl red.

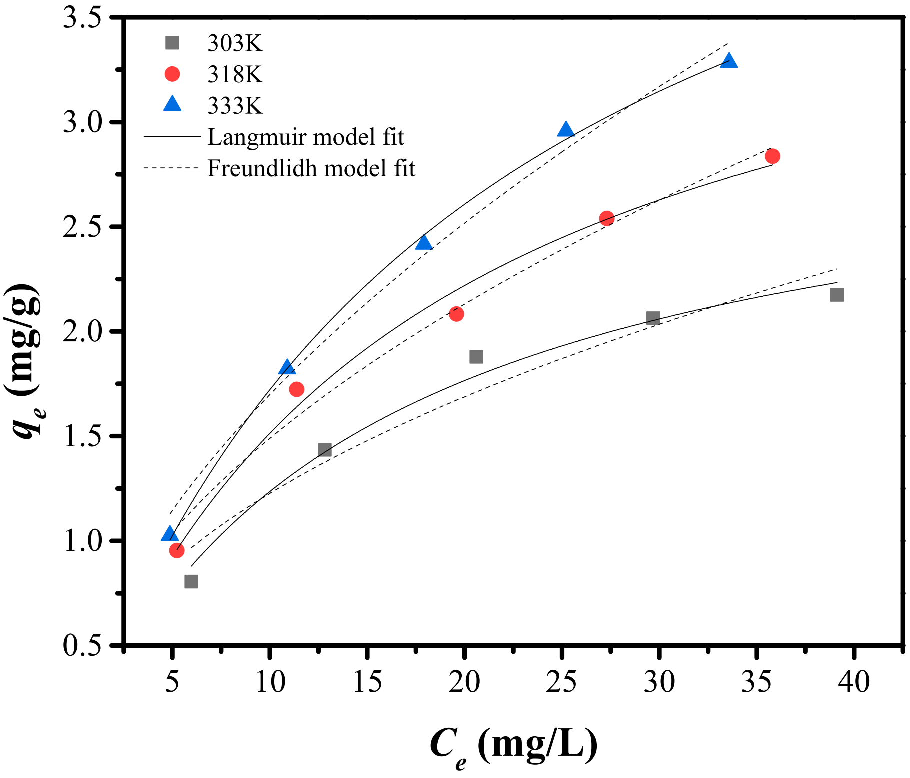

Figure S4. The adsorption isotherm measured for the adsorption system with the MIPMs-2 which was prepared in the second batch in order to evaluate the reliability of the fabrication method.

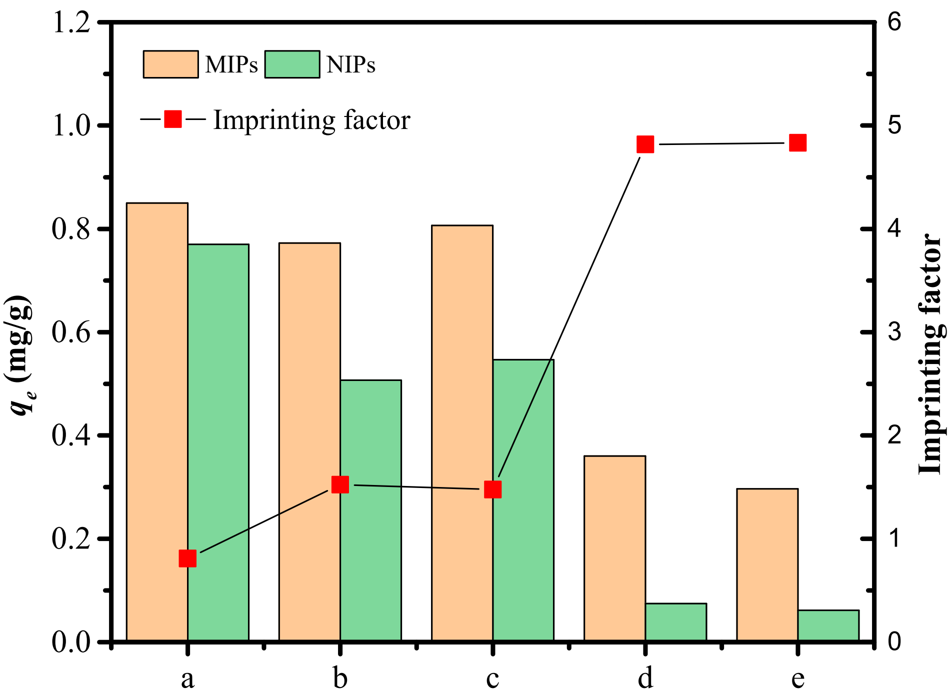

Figure S5. Investigation of the impact of the type of solution used as the medium for the adsorption experiment. The specific solution used as the media for the adsorption experiment is provided as follows: a—pure water; b—a water solution of ammonium acetate (4.6mmol/L); c—a water solution of Triton X-100 (4.6mmol/L); d—pure acetonitrile;e—an acetonitrile solution of ammonium acetate (4.6mmol/L).

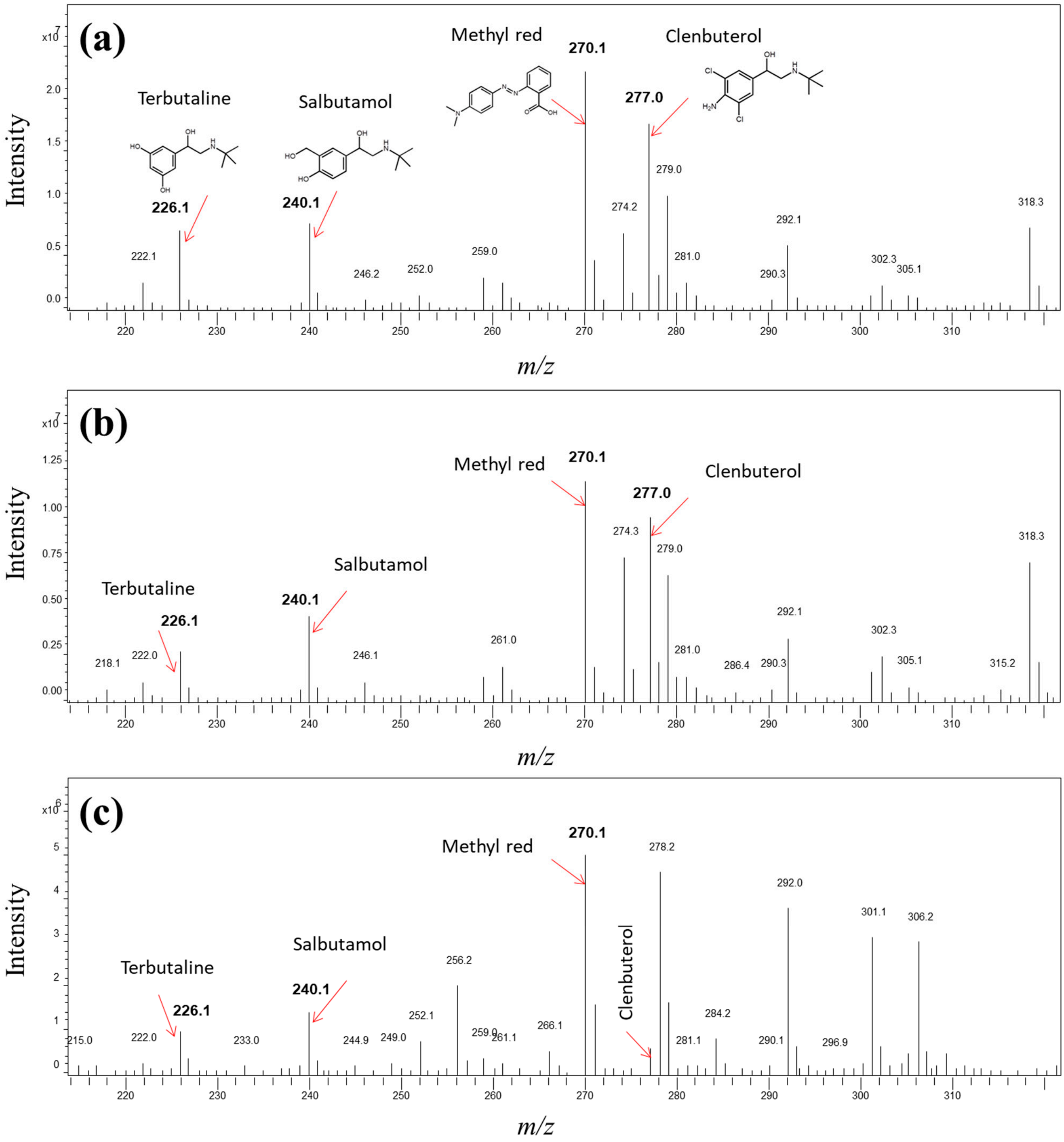

Figure S6. EIS-MS spectra of the mixed analyte solution with clenbuterol, methylene blue, terbutaline and Salbutamol before (a), and after processing with NIPMs (b) or with MIPMs (c).

Table S1. Pseudo-first-order and Pseudo-second-order kinetic parameters obtained via the adsorption of the clenbuterol onto MIPMs and NIPMs.

Adsorbents	Pseudo-first-order			Pseudo-second-order		
	q_{1e} (mg/g)	k_1 (1/min)	R^2	q_{2e} (mg/g)	k_2 (g/mg min)	R^2
**MIPMs**	0.8619	0.1649	0.9977	0.9726	0.02159	0.9795
**NIPMs**	0.6077	0.01171	0.9878	0.7074	0.01971	0.9908

Table S2. Langmuir and Freundlich parameters obtained via the adsorption of the clenbuterol onto MIPs and NIPs at different temperatures.

Adsorbents	T (K)	Langmuir model			Freundlich model		
		q_m (mg/g)	k_L (L/g)	R^2	k_F (L/g)	$1/n$	R^2
**MIPs**	303	3.735	0.05126	0.9991	0.3772	0.5244	0.9851
	318	4.338	0.05650	0.9981	0.4652	0.5203	0.9835
	333	5.182	0.05573	0.9981	0.5229	0.5399	0.9911
NIPs	303	2.701	0.03940	0.9836	0.2202	0.5513	0.9896
	318	2.711	0.05584	0.9860	0.3188	0.4870	0.9904
	333	4.142	0.03637	0.9879	0.2871	0.5906	0.9909

Table S3. Comparison of the isothermal adsorption of clenbuterol onto the molecularly imprinted samples (i.e., MIPMs and MIPMs-2) prepared in two different batches.

Adsorbents	T (K)	Langmuir model			Freundlich model		
		q_m (mg/g)	k_L (L/g)	R^2	k_F (L/g)	$1/n$	R^2
**MIPMs**	303	3.735	0.05126	0.9991	0.3772	0.5244	0.9851
	318	4.338	0.05650	0.9981	0.4652	0.5203	0.9835
	333	5.182	0.05573	0.9981	0.5229	0.5399	0.9911
**MIPMs-2**	303	3.090	0.06667	0.9861	0.4249	0.4604	0.94039
	318	4.160	0.05716	0.9900	0.4532	0.5166	0.98465
	333	5.370	0.04717	0.9987	0.4591	0.5679	0.99049

Table S4. Thermodynamic parameter obtained via the adsorption of clenbuterol onto MIPs at different temperatures.

C_0 (mg/L)	$\Delta G°$ (kJ/mol)			$\Delta H°$ (kJ/mol)	$\Delta S°$ (J/mol)
	303 K	318 K	333 K		
10	4.843	4.458	4.041	12.94	26.70
20	5.387	4.958	4.663	12.73	24.30
30	6.008	5.685	5.342	12.73	22.17
40	6.426	6.151	5.912	11.62	17.18
50	6.883	6.625	6.288	12.87	19.73

Table S5. Study on the impact of the type of solution as the media for the adsorption of clenbuterol onto the MIPMs and NIPMs.

	a	b	c	d	e
$q_{e,\ MIPMs}$ (mg/g)	0.85	0.77	0.81	0.36	0.30
$q_{e,\ NIPMs}$ (mg/g)	0.64	0.51	0.55	0.07	0.06
IF	1.33	1.52	1.48	4.82	4.83

a—pure water;

b—a water solution of ammonium acetate (4.6mmol/L);

c—a water solution of Triton X-100 (4.6mmol/L);

d—pure acetonitrile;

e—an acetonitrile solution of ammonium acetate (4.6mmol/L).

Table S6. Distribution coefficient and selectivity coefficient data obtained via the adsorption of clenbuterol onto NIPs and MIPs in the presence of different competing species including terbutaline, salbutamol and methyl red.

**Adsorbate**	**MIPs**			**NIPs**			
	C_e (mg/L)	k_d (L/g)	k	C_e (mg/L)	k_d (L/g)	k	k'
**Clenbuterol**	0.3699	0.8516		0.8204	0.1855		
**Terbutaline**	0.6341	0.2881	2.956	0.8489	0.1638	1.132	2.611
**Salbutamol**	0.6983	0.2756	3.090	0.7819	0.2695	0.6883	4.489
**Methyl red**	0.7591	0.1872	4.550	0.6625	0.1985	0.9346	4.869
